# Development of a dual-target measles virus PCR assay and testing trends at a national reference laboratory

**DOI:** 10.1128/jcm.01402-25

**Published:** 2025-12-18

**Authors:** Cole Anderson, Megha Rawal, Weston Hymas, Patricia Slev, Benjamin T. Bradley

**Affiliations:** 1 Department of Pathology, University of Utah 161530, Salt Lake City, Utah, USA; 2 ARUP Laboratories 33294, Salt Lake City, Utah, USA; St Jude Children's Research Hospital, Memphis, Tennessee, USA

**Keywords:** measles, PCR, assay development, vaccine strain, genotype A

## Abstract

In 2025, measles cases in the United States have reached their highest level since the disease was officially declared eliminated in 2000. Molecular assays may assist in the early diagnosis of measles and improve contact tracing efforts. In this manuscript, we describe the validation of a real-time PCR assay on the Hologic Panther Fusion Open Access system for the detection of measles virus (MeV) and differentiation between wild-type and vaccine strains. After implementation, we examined 3 months of clinical testing data to understand testing trends and utilization. Over the study period, a total of 525 tests from 491 patients were performed. MeV was detected in 16 specimens with 6 wild-type and 10 vaccine strain identifications. Children less than 10 years old constituted the largest proportion of tested individuals (54.3%) and vaccine strain detections (9/10, median age 1.2 years), while wild-type infections were observed in individuals aged 20–50 (6/6, median age 32.6 years). Those with vaccine strain detected had significantly higher Ct values for the pan-measles target versus wild-type infections (33.6 vs 28.3; *P*-value < 0.05). Only 4.2% of patients in our cohort received paired serologic and molecular measles testing. When paired data were available, PCR had a positive agreement of 25% and a negative agreement of 98% with IgM results. Molecular testing for MeV with the ability to differentiate wild-type strains from vaccine strains is a helpful tool in the response to measles re-emergence.

**IMPORTANCE:**

The 2025 U.S. measles outbreak comes at a challenging time for public health in America. As vaccine hesitancy increases and resources are withdrawn from national and state public health laboratories, historically low incidence diseases develop into nationwide outbreaks that require increased testing capacity. In response to this need, our national reference laboratory developed a measles PCR assay that allows for the detection and separation of vaccine from wild-type strains. The assay was launched on the Hologic Panther fusion system to improve throughput and reduce turnaround times. In this research article, we describe the design of our assay, validation results, and early clinical performance.

## INTRODUCTION

Measles was declared eliminated from the United States nearly 30 years ago, after sustained high two-dose coverage with the measles-mumps-rubella (MMR) vaccine was achieved. Since then, national vaccination rates have ranged between 90% and 93%, below the recommended 95% threshold for sustaining elimination, with considerable variation at the state and county levels (1). For example, in New York and Idaho, the two-dose MMR vaccine coverage rates during the 2023–2024 school year were 97.7% and 79.6%, respectively (2). Disruptions caused by the COVID-19 pandemic and an upsurge in vaccine misinformation and hesitancy have been attributed to declining vaccination rates. As a result, the United States has seen over 1,600 measles cases to date in 2025, the highest number since measles was declared eliminated in the United States in 2000 (3–5). Many of these cases have been associated with a large outbreak in Gaines County, Texas (6).

Clinically, measles presents as a febrile rash with cough, coryza, or conjunctivitis. In non-outbreak settings, these clinical findings have a low positive predictive value and can be difficult to distinguish from other common infections, thus highlighting the need for laboratory confirmation (7). The detection of measles virus-specific IgM antibodies is often used for confirming measles infection; however, certain performance limitations must be considered. There is known cross-reactivity of IgM between other common febrile rash illnesses, such as parvovirus B19, rubella, Epstein-Barr virus, and cytomegalovirus (8). In low-incidence settings, where measles has been eliminated, the positive predictive value of IgM serology is exceedingly low (9). Furthermore, a quarter of measles patients do not have detectable IgM antibodies within the first 72 h after rash onset, nor can it be used to distinguish between vaccine-induced rashes and wild-type infections. Considering these limitations, current Centers for Disease Control and Prevention (CDC) guidelines recommend PCR and IgM testing for diagnosis of acute measles (10).

Measles virus (MeV) RNA can be detected by reverse transcription real-time PCR (RT-PCR) from properly collected and transported respiratory and urine specimens, ideally obtained as early as possible after rash onset. One of the first published RT-PCR assays was a pan-measles assay that could detect but not differentiate between vaccine (genotype A) and wild-type strains (11). In regions that have eliminated endemic measles, the ability to distinguish wild-type infections from vaccine-induced rash is critical as vaccine strain MeV is not considered contagious and patients do not require airborne precautions. It is estimated that 5% of individuals will develop some form of rash following measles vaccination (12). Historically, separation of wild type from vaccine strain MeV was performed via Sanger sequencing (10). However, sequencing can take several days to perform and is only available through select public health laboratories and the CDC, thus limiting the clinical actionability of these results. Newer PCR assays with the ability to specifically detect the vaccine strain have since been developed. Roy and colleagues designed an assay (MeVA) that relies upon a 23-base sequence that is shared across all vaccine strains but differs by 1–5 nucleotides within the highly conserved N gene amino terminus of wild-type strains (13). A similar assay has also been described for use on the Hologic Panther Fusion System, which is a fully automated, random access, and continuous loading system with a run time of approximately 3 h (14). Platforms that support high-throughput random-access testing improve turnaround times and support surge testing requirements often seen in outbreak settings (15).

In this study, we describe the analytical validation of a qualitative, dual-target RT-PCR assay (dt-MeV) that utilizes the Hologic Panther Fusion Open Access channel to detect MeV RNA with the ability to distinguish between vaccine and wild-type strains. We further examine clinical testing data and test utilization during the first 3 months of implementation at a national reference laboratory.

## MATERIALS AND METHODS

### Primers and probes

Primer and probe sequences from previously published assays were used to detect measles virus and the vaccine strain (11, 13) (Table S1). The Hummel assay, referred to as the MeV target, will detect both vaccine and wild-type strains. The Roy assay, referred to as the MeVA target, is specific for vaccine strain only. Hologic internal control primers and probes were used to monitor for sample inhibition. Samples were reported as “measles virus detected” when the MeV (pan-measles) target was detected and the MeVA (vaccine) target was not detected. For purposes of this study, we categorized any MeV+/MeVA− samples as positive for wild-type measles, though this result could also be observed for vaccine strain samples with low viral load. Samples were reported as “measles virus, vaccine strain detected” when both the MeV and MeVA targets were detected. Interpretive result comments are provided in Table S2.

### Validation testing

Sample preparation and RNA extraction were performed as previously described for the Hologic Panther Fusion System (14). Optimized reagent concentrations and thermocycling conditions are shown in Tables S1 and S3. Limit of detection (LoD) and accuracy were determined using positive culture material for vaccine strain and genotype B3 (wild type) which were quantified by droplet-digital PCR. Fivefold serial dilutions of the quantified cultures were made in VTM and urine samples. The lowest concentration where all six replicates amplified was established as the limit of detection, which was confirmed by performing an additional 20 replicates. For the vaccine strain, LoD determination required positive detection in both MeV and MeVA channels. To assess accuracy, a total of 80 samples—40 respiratory swabs (15 vaccine strain positive, 15 wild-type positive, and 10 negative) and 40 urine samples (15 vaccine strain positive, 15 wild-type positive, and 10 negative)—were tested. Performance was evaluated at low (10× LoD, *n* = 10), medium (100× LoD, *n* = 3), and high (1,000× LoD, *n* = 2) analyte concentrations. To evaluate the specificity of MeV and MeVA primers/probes, *in silico* analysis was performed against 18 clinically relevant pathogens. Previously tested clinical samples and control material were also used to evaluate cross-reactivity. Six different measles genotypes (D4, D8, D9, G3, H1, and B3) from cell culture material were tested to evaluate inclusivity (provided courtesy of Dr. Ryan Relich of Indiana University).

### Clinical data

Clinical data were obtained from an in-house database that included PCR and IgM/IgG results and patient demographics. For serological testing, measles IgG was performed by a chemiluminescent immunoassay (Diasorin Inc., Stillwater, MN), while IgM was performed by enzyme-linked immunosorbent assay (Awareness Technology, Quest International, Doral, FL). This study was performed under University of Utah IRB00007275.

### Statistical analysis

Graphing and related statistics were performed using GraphPad Prism (Version 10.5.0). Turnaround times, defined as time from sample receipt to reported result, were analyzed using Tukey’s multiple comparisons test. Probit analysis was conducted to determine LoD using MedCalc (Version 23.1.7). Continuous variables were analyzed using the Mann-Whitney test, and data are reported as medians and 25%–75% interquartile ranges (IQRs).

## RESULTS

Validation studies of the dt-MeV assay demonstrated acceptable performance characteristics. The estimated LoD in copies/mL for genotype B3 (wild type) and vaccine strain (genotype A) in respiratory samples was 3,039.8 (95% CI: 1,722.0–10,634.9) and 689.8 (95% CI: 398.5–2,178.2), respectively, as calculated by Probit analysis (Table 1; Fig. S1). Similar LoDs were observed using a manual approach with nucleic acid extraction on the Chemagic MSM I (Perkin Elmer) and amplification on the QuantStudio 12K Flex (Thermo Fisher Scientific) RT-PCR instrument (Table S4). All contrived specimens with spiked-in vaccine and wild-type strains at various multiples of the LoD (10–1,000×) were detected by the dt-MeV assay for an analytical sensitivity of 100% (Table S5). Similarly, measles RNA was not detected in negative specimens, and cross-reactivity was not observed when tested against 18 clinically relevant viruses (Table S6). Inclusivity was assessed against six wild-type genotypes (D4, D8, D9, G3, H1, and B3), including genotype D8 which has been responsible for the majority of U.S. measles cases in 2025. All wild-type isolates were appropriately detected in the MeV channel and not detected in the MeVA channel.

**TABLE 1:** Limit of detection

				Mean Ct			
Analyte	Specimen type	Copy no./mL	Positive results/total tested	MeV	MeVA	LoD copies/mL (copies/reaction)	95% CI (copies/mL)
Vaccine (genotype A)	Respiratory	20,000	6/6	32.6	34.1	689.8 (10.2)	398.5–2,178.2
		4,000	6/6	34.5	37.9		
		800	25/26	39.5	40.8		
		160	3/6	39.7	41.0		
		32	0/6	–^*a*^	–		
		6.4	0/6	–	–		
Wild type (genotype B3)	Respiratory	62,500	6/6	35.3	–	3,039.8 (44.9)	1,722.0–10,634.9
		12,500	6/6	38.4	–		
		2,500	25/26	41.1	–		
		500	0/6	–	–		
		100	1/6	44.6	–		
		20	0/6	–	–		
Vaccine (genotype A)	Urine	4,000	6/6	36.5	37.3	743.6 (11.0)	
		800	25/26	39.4	41.2		488.5–1,705.8
		160	1/6	39.6	42.5		
		32	0/6	40.9	–		
		6.4	0/6	–	–		
		1.28	0/6	–	–		
Wild type (genotype B3)	Urine	12,500	6/6	38.8	–	2,323.6 (34.3)	1,526.5–5,330.5
		2,500	25/26	41.8	–		
		500	1/6	42.3	–		
		100	0/6	–	–		
		20	0/6	–	–		
		4	0/6	–	–		

^*a*^ "–" indicates that no value was generated for this target.

**Fig 1:**
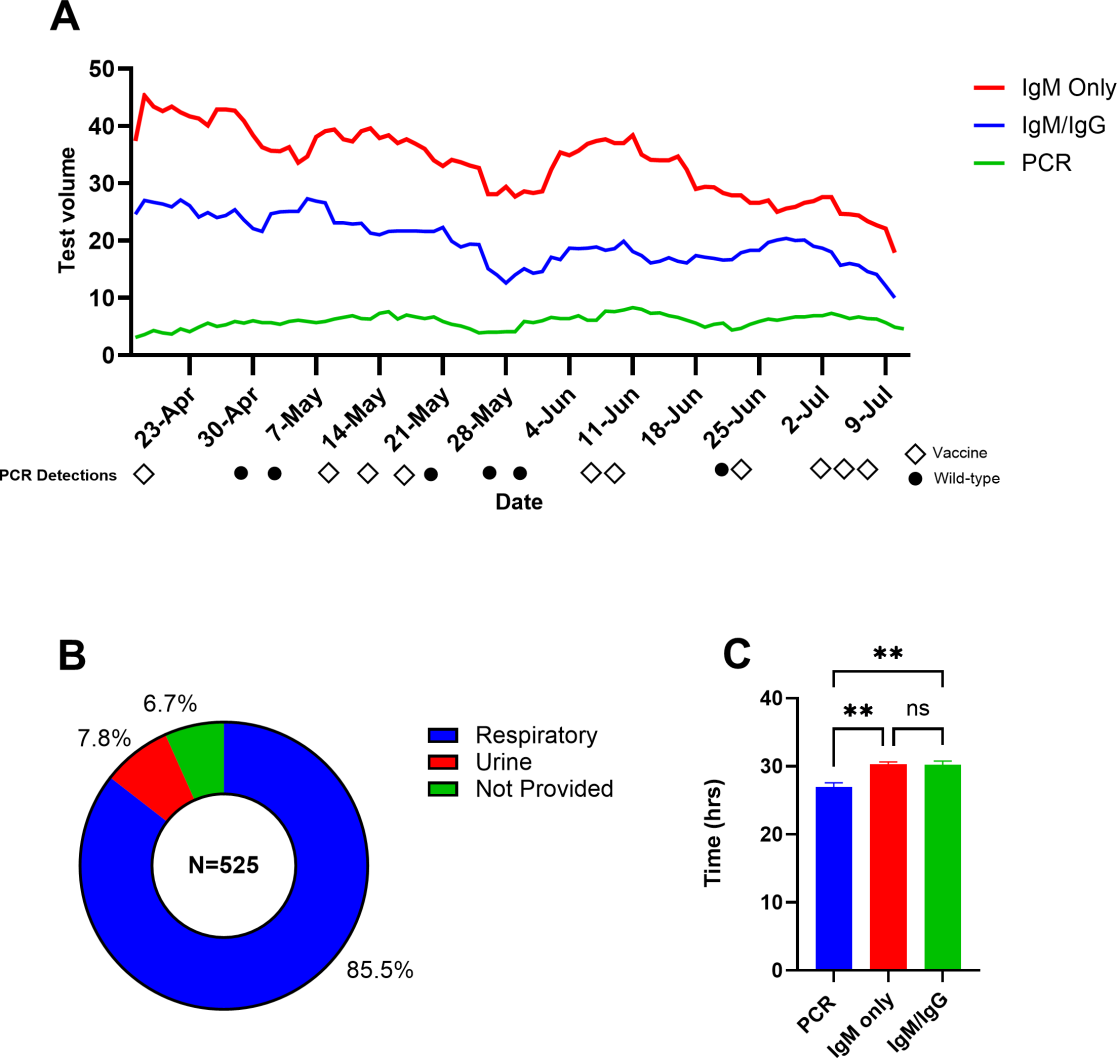
Three-month test utilization. (**A**) Rolling 7-day average test volume for measles PCR, IgM alone, and IgM/IgG combination. Positive PCR detections during the first 3 months of implementation shown below the *x*-axis. (**B**) Distribution of specimen types submitted for measles PCR. (**C**) Average turnaround time in hours for measles PCR, IgM alone, and IgM/IgG combination. **P < 0.01 by Tukey’s multiple comparisons test. The figure demonstrates testing performed at ARUP Laboratories. Additional clinical testing for measles may have been performed at outside institutions or public health laboratories. The clinical indication for IgM serology was not provided.

From April to July 2025, 525 measles PCR tests were ordered for 491 patients (Fig. 1A). Daily PCR test volume averaged 5.7 (range, 1–14) tests per day during this period, while combined IgM only and IgM/IgG averaged 51.7 (range, 0–110) tests per day. Over 85% (*n* = 449) of the specimens submitted for PCR were from a respiratory source (Fig. 1B). In 14 patients, urine was the only specimen submitted, while 27 patients had both urine and respiratory samples tested. The average turnaround time (in lab to verified result) for measles PCR and IgM was 27.0 and 30.3 h, respectively (Fig. 1C).

**Fig 2:**
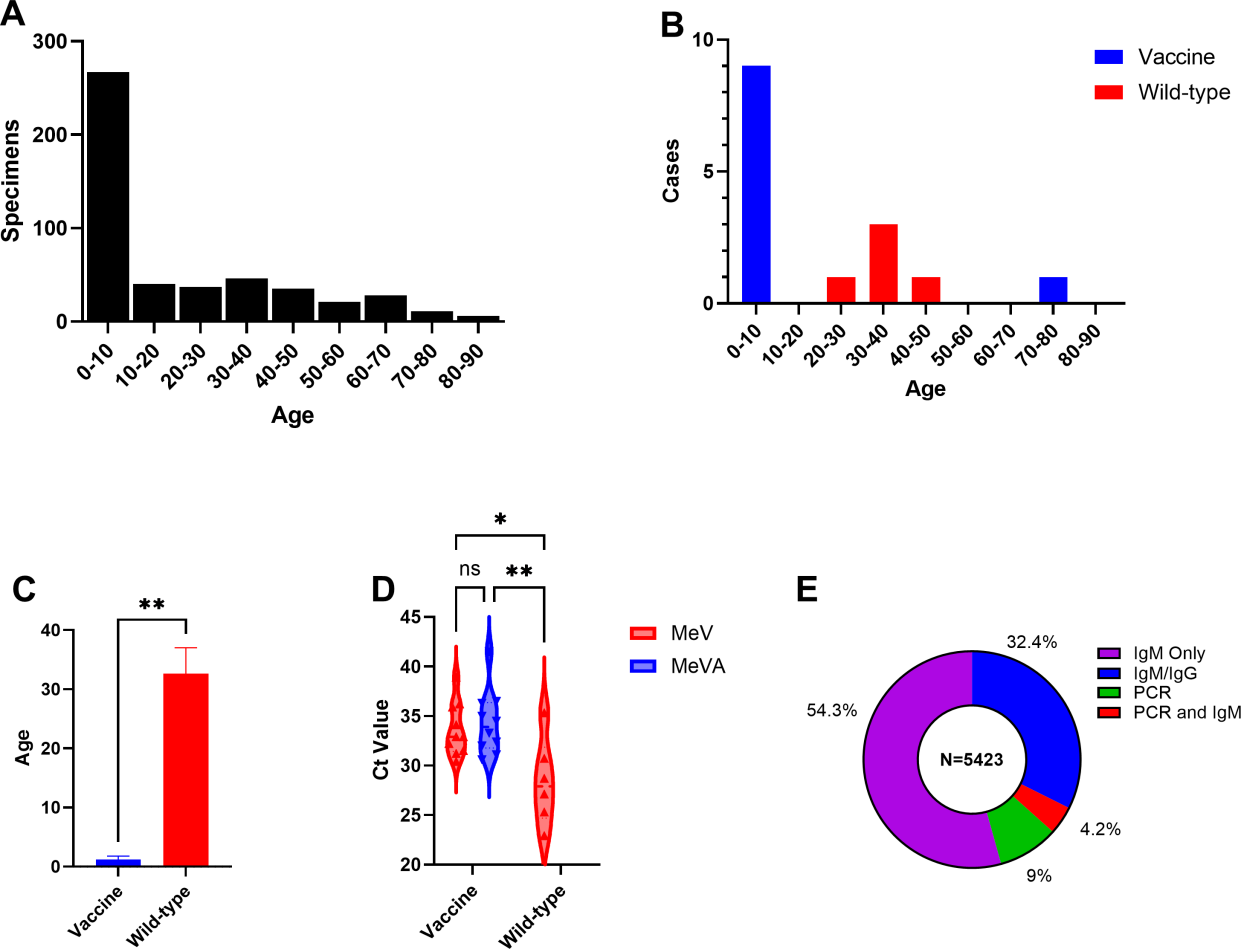
Measles PCR test characteristics. (**A**) Age distribution of all patients with measles PCR. (**B**) Age distribution of vaccine and wild-type measles cases. (**C**) Median age of vaccine-associated and wild-type cases. (**D**) Violin plot of Ct values between vaccine-associated and wild-type measles cases. (**E**) Three-month distribution of measles testing by diagnostic modality. *P < 0.05, **P < 0.01 and n.s, not statistically significant by Mann-Whitney test. The figure demonstrates testing performed at ARUP Laboratories. Additional clinical testing for measles may have been performed at outside institutions or public health laboratories. The clinical indication for IgM serology was not provided.

Of the individuals tested by PCR, 54.3% (*n* = 267) were 0–10 years old, including 20.6% (*n* = 101) aged 0–1 years old (Fig. 2A). Other age groups ranged from 1.2% to 9.4%. Measles was detected in 16 patients by PCR. The vaccine strain was detected in 10 of these patients, while wild-type virus was detected in 6. Invalid results due to internal control failure occurred in eight patients (1.5% of testing volume). Of the vaccine-associated cases, 90% (9/10) were identified in 0–10 year olds with a single vaccine case identified in an individual 70–80 years old (Fig. 2B). All wild-type cases were in patients 20–50 years old (*n* = 6). The median age for vaccine-associated cases was 1.2 (IQR 1.1, 1.8) years, while the median age for wild-type cases was 32.6 (IQR 16.7, 37.0) years (Fig. 2C). Cycle threshold (Ct) values for the pan-MeV target were significantly higher in vaccine cases than in wild-type cases (33.6 vs 28.3; *P*-value < 0.05) (Fig. 2D). For vaccine-associated cases, the mean MeVA Ct was slightly higher than the MeV Ct, though this did not reach statistical significance (31.7 vs 31.0; n.s.).

Data were collected regarding potential measles diagnostic testing (IgM, IgM/IgG, and PCR) performed by our laboratory during the 3-month study period. Overall, IgM alone was the most commonly ordered test during this period (54.3%, *n* = 2,946), followed by IgM/IgG in combination (32.4%, *n* = 1,756). PCR constituted a minority of the overall patient testing volume at 9% (*n* = 491) with only 4.2% (*n* = 230) of patients in our cohort receiving orders for both serology (IgM ± IgG) and PCR (Fig. 2E). Of patients with both PCR and IgM results, 88% (*n* = 202) were negative. Both measles RNA and IgM were detected in six patients (three vaccine and three wild type), while four patients with negative IgM had detectable measles RNA (three vaccine and one wild type) (Table 2). Measles RNA was not detected in 18 patients with positive IgM serology (one invalid in a patient who received the MMR vaccine 1 month prior to sample collection) (Table S7). When compared to IgM serology, measles PCR had a positive and negative percent agreement of 25% (95% CI: 0.12–0.45) and 98% (95% CI: 0.95–0.99), respectively.

**TABLE 2:** Patients with positive measles PCR

Patient no.	State	Age (yr)	Gender	Strain	MeV Ct	MeVA Ct	IgM*^a^* (AU)	IgG*^b^* (AU/mL)
1	TX	1	M	Vaccine	32.2	32.4	7.08	<5.0
2	TX	<1	F	Vaccine	31.2	31.1	9.21	56.9
3	IL	1	F	Vaccine	32.9	35.0	0.68	N/A^*d*^
4	UT	1	F	Vaccine	35.9	36.3	2.92	<5.0
5	UT	2	F	Vaccine	31.5	32.0	0.20	<5.0
6	ID	1	M	Vaccine	36.2	36.5	0.94	<5.0
7	OH	1	F	Vaccine	41.5	38.9	N/A	N/A
8	OH	2	F	Vaccine	34.5	34.1	N/A	N/A
9	UT	1	F	Vaccine	33.3	32.9	N/A	N/A
10	UT	76	F	Vaccine	30.6	30.4	N/A	N/A
11	TX	34	F	Wild type	28.7	–^*c*^	6.23	N/A
12	CO	31	M	Wild type	35.3	–	1.95	N/A
13	CO	35	M	Wild type	22.9	–	10.54	N/A
14	UT	21	M	Wild type	27.1	–	0.28	<5.0
15	TX	5	F	Wild type	25.3	–	N/A	N/A
16	ID	43	M	Wild type	30.7	–	N/A	N/A

^*a*^ Awareness Technology: 1.21 AU or greater is positive.

^*b*^ Diasorin Inc.: 16.5 AU/mL or greater is positive.

^*c*^ "–" indicates that no value was generated for this target.

^*d*^ "N/A" indicates that the test was not performed for this patient sample.

## DISCUSSION

The public health response to measles outbreaks requires considerable resources to quickly identify exposed cases and to initiate MMR vaccination campaigns in non-immune populations. The dt-MeV assay presented in this study is a rapid, highly sensitive, and specific assay to detect and differentiate vaccine-associated and wild-type measles strains. The limit of detection between the vaccine strain and genotype B3 (wild type) was within 1 log_10_ (689.8 vs 3,039.8 copies/mL, respectively). We did note that Ct values were slightly higher for the MeVA target versus the MeV target. This replicates prior studies which found that the MeV target may be 10-fold more sensitive as compared to the MeVA target (11, 13). This can potentially lead to a false-positive wild-type result when testing a specimen with a low concentration of vaccine strain virus if using our reporting criteria. For example, we identified a vaccine strain case with a subtle MeVA signal that was initially called “Not Detected” by the instrument software but on repeat testing generated a “Detected” result (Fig. S2). The patient in this case had received their first MMR dose 11 days prior to specimen collection. Immunization history was also available for two additional vaccine cases where patients had received the MMR vaccine at 11 and 39 days before specimen collection. The clinical impact of misidentifying a vaccine strain isolate as wild type could lead to unnecessary patient isolation and contact tracing (9, 11).

During the first 3 months of clinical testing, measles virus was detected in 16 patients with 10 vaccine-associated and six wild-type cases reported. Given the MMR vaccine schedule begins at 12 months, it is unsurprising that the majority (9/10) of vaccine detections occurred in children 0–1 years old with a median age of 1.2 years. This is in comparison to the wild-type cases which were detected at a median of 32.6 years. Interestingly, a single vaccine-associated case in the 70- to 80-year-old cohort occurred in an immunocompromised patient who received the MMR vaccine over 1 month before testing and subsequently developed severe pneumonia. In this case, the measles virus vaccine strain was detected from the patient’s nasopharyngeal and bronchoalveolar lavage specimens. The MMR vaccine is contraindicated in immunosuppressed individuals, and vaccination may lead to pneumonia, encephalitis, or death (16).

In vaccine-associated cases, the MeV Ct value was significantly higher than that in wild-type cases, which is consistent with previously published reports on vaccine-associated cases. In an Ohio outbreak in 2022, Washam et al. reported a median Ct of 33.7 for vaccine cases versus 19.0 for wild-type cases (17). In our study, we saw similarly high MeV Ct values for vaccine cases at 34, but the difference when compared to MeV Ct values for wild-type cases, while statistically significant at Ct 27.8, was not as pronounced as the Washam study. One potential explanation for this is that our cohort included previously vaccinated patients who were experiencing breakthrough wild-type infections due to secondary vaccine failure, where protective immunity wanes over time. In these patients, clinical presentation is often mild, and measles RNA can be detected in the setting of an IgG response with a transient or absent IgM response (18). Studies suggest that individuals experiencing breakthrough measles infections will have higher Ct values (19). While vaccination histories were not available for all the wild-type infections in our cohort, in discussions with the treating physician, at least two were confirmed to have been vaccinated.

Measles vaccination is recommended as post-exposure prophylaxis for unvaccinated children who have been exposed to measles virus. Therefore, it is theoretically possible that a co-infection with vaccine and wild-type measles virus could occur. While no documented co-infections have been reported, our dt-MeV assay is not designed with a specific wild-type target. In the event of a co-infection, the sample would be reported as “Measles virus vaccine strain detected” based on positive MeV and MeVA signals. However, experimental mixing studies with vaccine and wild-type measles virus have shown that the difference between MeVA and MeV Ct values can potentially help identify co-infected patients. Specifically, a Ct difference of 3.54 between MeVA and MeV was shown to have a sensitivity and specificity of 90% and 98%, respectively, in identifying co-infections (20).

Current CDC guidelines mention both molecular and serologic (IgM or IgM/IgG) testing as tools for the diagnosis of acute measles. In our study, we found limited PCR testing relative to serology. Over 85% of patients received IgM testing alone or in combination with IgG during the 3 months in which PCR was available. A minority of patients, 4.2%, had both PCR and IgM testing ordered at our laboratory. While we did not receive the clinical indication for testing, these data may suggest underutilization of PCR testing during our study. Reasons may include limited access during the first months of availability as the assay required client sites to build an orderable test in their electronic medical record, use a “miscellaneous” test order, or complete a faxed test requisition. Further, we cannot exclude that physician awareness of this testing option is low due to molecular measles testing being historically available only through public health laboratories and the CDC. The rapid turnaround time for our dt-MeV assay at 27 h was faster than serology and argues that complete diagnostic testing for measles can quickly be achieved during outbreaks.

There are several limitations of this study. As a national reference laboratory, we only had access to test results that were performed within our system. It is likely that patients included in this study had additional testing elsewhere and that the overall rate of co-testing by PCR and IgM was higher than reported here. Over one third of the IgM orders placed were part of a combined IgM/IgG order set. In these cases, the intent may have been to assess vaccination status rather than diagnose acute measles, even though IgM testing is not recommended for evaluating vaccination status. Furthermore, we did not have access to clinical or vaccination histories to help adjudicate PCR negative/IgM positive results. We did not place any restrictions on acceptable patient specimens and therefore may have experienced a higher number of vaccine detections as compared to public health laboratories, which often require an evaluation of epidemiological risk prior to measles testing. In the latter situation, inclusion of the MeVA target may be less essential as part of the testing algorithm.

Our study highlights the inherent difficulties commercial, reference, and clinical laboratories face in responding to emerging and re-emerging pathogens. One is a lack of clinical isolates for assay development and validation. We relied on contrived samples using cultured isolates during our validation as clinical isolates of measles were not readily available. This is a familiar challenge faced during early development efforts for SARS-CoV-2, monkeypox virus, and influenza A(H5) viruses (21–23). Second is a lack of confirmatory sequencing data from the positive cases we reported. Specimens positive for wild-type measles were sent to their respective state public health laboratories for confirmatory testing. Of the six wild-type cases, sequencing (genotype D8) data were returned for one of these cases, and PCR confirmation was returned for another. These limitations argue for increased interoperability between clinical and public health laboratories through sharing of clinical isolates for assay development and exchange of confirmatory testing data to help monitor assay performance.

In summary, we demonstrate the feasibility and clinical implementation of an automated dual-target, RT-PCR assay for the detection and differentiation of wild-type and vaccine strain measles virus. Laboratories interested in developing a similar assay should focus on stewardship to reduce unnecessary testing in recently vaccinated populations and build provider awareness about the availability and accuracy of molecular testing.

## SUPPLEMENTAL MATERIAL

Supplemental tables and figures jcm.01402-25-s0001.docx Tables S1 to S7, and Figures S1 and S2.

ASM does not own the copyrights to Supplemental Material that may be linked to, or accessed through, an article. The authors have granted ASM a non-exclusive, world-wide license to publish the Supplemental Material files. Please contact the corresponding author directly for reuse.
