# Supplementary material for: Development of a dual-target measles virus PCR assay and testing trends at a national reference laboratory
Source: J Clin Microbiol. 2025 Dec 18;64(1):e01402-25. doi: 10.1128/jcm.01402-25 (PMC12802152; doi:10.1128/jcm.01402-25)
Supplement: Supplemental tables and figures — Tables S1 to S7, and Figures S1 and S2. [file jcm.01402-25-s0001.docx]

**TABLE S1** Final concentrations of PCR reagents

| **Reagent** | **Concentration (µM)** |
| --- | --- |
| 1M MgCl_2_ | 4,000 |
| 1M KCL | 5,000 |
| 1M Tris, pH 8.0 | 5,000 |
| MeV F: 5’-TGGCATCTGAACTCGGTATCAC-3’ | 0.4 |
| MeV R: 5’-TGTCCTCAGTAGTATGCATTGCAA-3’ | 0.4 |
| MeV Probe: 5’-HEX-CCGAGGATGCAAGGCTTGTTTCAGA-BHQ1-3’ | 0.2 |
| MeVA F: 5’-AGGATGAGGCGGACCAATACTT-3’ | 0.8 |
| MeVA R: 5’GAACCATCCGAACCTGGAT-3’ | 0.8 |
| MeVA Probe: 5’-FAM-CATGATGATCCAATTAGTAGTGA-BBQ-3’*^a^* | 0.2 |
| Internal Control Primer *^b^* | 0.4 |
| Internal Control Probe *^b^* | 0.2 |

*^a^* Underlined characters indicate locked nucleic acid bases.

*^b^* Sequences not available due to proprietary restrictions (Hologic Inc.)

**TABLE S2** Interpretive comments

| **Measles Target** | |  |
| --- | --- | --- |
| **MeV** | **MeVA** | **Result** |
| Positive | Negative | Measles virus: Detected. Specimens with low levels of vaccine strain may be reported as wild-type due to the lower sensitivity of the vaccine-specific target (MeVA) compared with the pan-measles target (MeV) |
|  |  | Measles virus, vaccine strain: Not Detected |
|  |  |  |
| Positive | Positive | Measles virus: Not Detected |
|  |  | Measles virus, vaccine strain: Detected. These results are consistent with the detection of vaccine strain (genotype A) measles virus and may indicate recent vaccination. |
|  |  |  |
| Negative | Positive | Sample sent to Medical Director for review. |
|  |  |  |
| Negative | Negative | Measles virus: Not Detected |
|  |  | Measles virus, vaccine strain: Not Detected |

**TABLE S3** PCR cycling conditions

| **Step** | **Temperature (°C)** | **Time (mm:ss)** |
| --- | --- | --- |
| 1 | 46 | 08:00 |
| 2 | 95 | 02:00 |
| 3 | 95 | 00:05 |
| 4 | 60 | 00:22 |
| Repeat steps 3-4 for 45 cycles  MeVA threshold: 800 RFU*^a^*  MeV threshold: 1000 RFU | | |

*^a^* Relative fluorescence units

**TABLE S4** Limit of detection comparison between Hologic and QuantStudio RT-PCR platforms

|  |  | **Hologic** | | **QuantStudio** | |
| --- | --- | --- | --- | --- | --- |
| **Assay** | **Specimen type** | **LoD (copies/mL)** | **95% CI (copies/mL)** | **LoD (copies/mL** | **95% CI (copies/mL)** |
| Vaccine | Respiratory | 689.8 | 398.5-2178.2 | 470.8 | 189.6-81185.4 |
| Wild-type | Respiratory | 3039.8 | 1722.0-10634.9 | 2953.6 | 1113.6-109366.0 |

**TABLE S5** Analytical sensitivity and specificity of the dt-MeV assay

|  |  |  |  | **Mean Ct** | |
| --- | --- | --- | --- | --- | --- |
| **Strain** | **Specimen type** | **LoD** | **No. with positive results/total no. tested** | **MeV** | **MeVA** |
| Vaccine | Respiratory | 10x | 10/10 | 33.8 | 35.8 |
|  |  | 100x | 3/3 | 31.5 | 32.5 |
|  |  | 1,000x | 2/2 | 28.6 | 29.2 |
| Wild-type | Respiratory | 10x | 10/10 | 33.0 | - |
|  |  | 100x | 3/3 | 30.3 | - |
|  |  | 1,000x | 2/2 | 25.6 | - |
| Vaccine | Urine | 10x | 10/10 | 33.8 | 36.2 |
|  |  | 100x | 3/3 | 33.8 | 33.0 |
|  |  | 1,000x | 2/2 | 28.8 | 30.6 |
| Wild-type | Urine | 10x | 10/10 | 33.1 | - |
|  |  | 100x | 3/3 | 29.9 | - |
|  |  | 1,000x | 2/2 | 24.6 | - |
| Negative | Respiratory | - | 0/10 | - | - |
| Negative | Urine | - | 0/10 | - | - |

**TABLE S6** Specificity of the dt-MeV assay

|  | **Ct Value** | |
| --- | --- | --- |
| **Pathogen** | **MeV** | **MeVA** |
| Mumps | - | - |
| Poliovirus | - | - |
| Echovirus | - | - |
| Parvovirus | - | - |
| HHV 6A | - | - |
| HHV 6B | - | - |
| Influenza A | - | - |
| Influenza B | - | - |
| HSV-1 | - | - |
| HSV-2 | - | - |
| CMV | - | - |
| BK virus | - | - |
| Enterovirus D68-2 | - | - |
| RSV | - | - |
| Adenovirus, HMPV, Rhinovirus | - | - |
| Parainfluenza virus 1-4 | - | - |
| Measles D4 | 24.5 | - |
| Measles D8 | 25.4 | - |
| Measles D9 | 22.9 | - |
| Measles G3 | 20.9 | - |
| Measles H1 | 26.4 | - |
| Measles B3 | 20.6 | - |

**TABLE S7** PCR negative with positive IgM

| **Patient no.** | **State** | **Age (yrs)** | **Gender** | **IgM (AU)*^a^*** | **IgG (AU/mL)*^b^*** |
| --- | --- | --- | --- | --- | --- |
| 1 | TX | 41 | M | 1.95 | 193.0 |
| 2 | TX | 1 | M | 5.83 | 72.0 |
| 3 | TX | <1 | F | 1.53 | >300.0 |
| 4 | NE | 33 | F | 6.00 | 288.0 |
| 5 | UT | 5 | M | 2.56 | >300.0 |
| 6 | OH | 14 | M | 1.30 | 127.0 |
| 7 | ID | <1 | F | 11.57 | >300.0 |
| 8 | UT | <1 | F | 2.55 | >300.0 |
| 9 | TX | 9 | F | 2.72 | N/A |
| 10 | IL | 18 | F | 5.51 | N/A |
| 11 | IL | 6 | M | 1.56 | N/A |
| 12 | TX | 63 | M | 1.83 | N/A |
| 13 | CO | 35 | F | 3.53 | N/A |
| 14 | NE | 7 | F | 2.75 | N/A |
| 15 | FL | 45 | M | 1.26 | N/A |
| 16 | TX | 24 | M | 5.51 | N/A |
| 17 | CO | 2 | F | 1.22 | >300.00 |
| 18*^c^* | UT | 1 | M | 5.70 | >300.00 |

*^a^*  Awareness Technology, 1.21 AU or greater is positive

*^b^* Diasorin Inc., 16.5 AU/mL or greater is positive

*^c^* Invalid PCR due to internal control failure

**Figure S1.** Probit analysis. Probit analysis of LoD for wild-type and vaccine measles virus in respiratory and urine matrices. The x-axis represents viral RNA copies/mL, and the y-axis indicates the probability of detection. The LoD is defined as the viral RNA concentration corresponding to a 95% detection probability. The red circles are data points that represent the observed detection rates for each dilution, and the fitted probit curves represent the statistical modelling of detection probabilities. Shaded areas show the 95% CI around the probit curves.

**Figure S2.** Amplification plots of a vaccine-associated measles case. (A) Plot showing amplification of the pan-measles (MeV) (Ct = 39.6) and internal control (IC) targets. The vaccine target (MeVA) shows a subtle increase in fluorescence but did not meet the positivity criteria of 800 relative fluorescence units. This initial result was sent for Medical Director review and repeat analysis. (B) Repeat analysis showing amplification of MeV (Ct = 35.9), MeVA (Ct = 36.3), and IC targets. The patient had received their first MMR dose 11 days prior to specimen collection, and the case was reported as measles vaccine strain detected.
